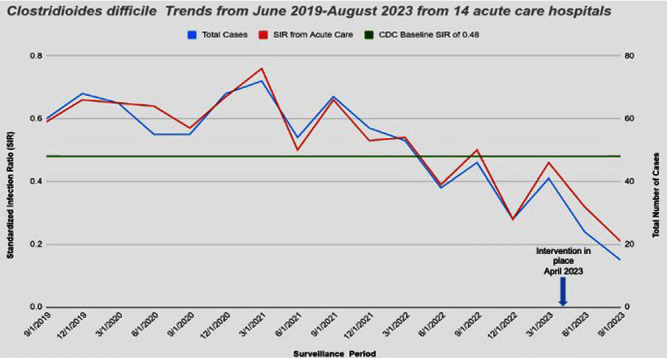# Breaking the Reflex: Impact in Hospital-Acquired Infection Incidence for Clostridioides difficile Infection

**DOI:** 10.1017/ash.2024.189

**Published:** 2024-09-16

**Authors:** Mamta Sharma, Reese Cosimi, Alysia Stewart, Nomides Nicole, Lisa Sturm, William Hart, Leonard Johnson

**Affiliations:** Ascension St. John Hospital; Ascension; Ascension Michigan

## Abstract

**Background:** Nucleic acid amplification tests (NAAT) do not distinguish between colonization and Clostridioides difficile (C.diff) associated diarrhea. On April 5th 2023 our laboratory introduced a new C. diff testing methodology. Previously, if a C. diff screen result was negative for toxin and positive for glutamate dehydrogenase (GDH), a second confirmatory test was conducted with NAAT. This confirmatory test was removed from our testing algorithm. NAAT testing may be ordered ad hoc when clinically relevant diarrhea persists, and alternative etiologies have been excluded. We wanted to evaluate the impact of change with testing methods. **Method:** Retrospective review of all inpatient hospital-acquired C.diff infections reported to NHSN database from Ascension Michigan Market which comprises 14 acute care hospitals from June 2019 to August 2023. Data for C diff was analyzed every quarter. The risk adjustments used to calculate the Standardized Infection Ratios (SIRs) for C. diff infections was set at 0.48 based on CDC mean SIR established for acute care hospitals in 2022. **Results:** A total of 14 acute care hospitals were included from which 866 C.diff cases were reported during this period. Overall, the SIR dropped from 0.59 from June-August 2019 to 0.32 reported from March-May 2023; 45.7 % decrease. The maximum reduction in SIR was seen post intervention at 0.21 from June-August 2023 which was 78.3% below the benchmark of 0.48. (Figure) **Conclusions:** Strategies to optimize current laboratory tests are critical to differentiate C. diff infection from colonization. The current strategy by changing the testing method led to substantial reduction in C-diff. Diagnostic stewardship studies should ideally include outcome measures targeted to post-intervention patients to determine clinical relevance and patient safety. Optimizing test utilization remains a critical component of quality healthcare delivery. Future NHSN updated surveillance definition will require incorporating clinical decision-making into the metric; that is including a combination of any positive C-diff test plus initiation of antibiotic therapy for C-diff.

**Disclosure:** Reese Cosimi: Advisory Board - Abbvie

**Figure:**